# An EvoDevo Study of Salmonid Visual Opsin Dynamics and Photopigment Spectral Sensitivity

**DOI:** 10.3389/fnana.2022.945344

**Published:** 2022-07-11

**Authors:** Mariann Eilertsen, Wayne Iwan Lee Davies, Dharmeshkumar Patel, Jonathan E. Barnes, Rita Karlsen, Jessica Kate Mountford, Deborah L. Stenkamp, Jagdish Suresh Patel, Jon Vidar Helvik

**Affiliations:** ^1^Department of Biological Sciences, University of Bergen, Bergen, Norway; ^2^Umeå Centre for Molecular Medicine, Umeå University, Umeå, Sweden; ^3^School of Life Sciences, College of Science, Health and Engineering, La Trobe University, Melbourne, VIC, Australia; ^4^Institute for Modeling Collaboration and Innovation (IMCI), University of Idaho, Moscow, ID, United States; ^5^Lions Eye Institute, University of Western Australia, Perth, WA, Australia; ^6^Department of Biological Sciences, University of Idaho, Moscow, ID, United States; ^7^Institute for Bioinformatics and Evolutionary Studies, University of Idaho, Moscow, ID, United States

**Keywords:** photoreception, eye, atomistic molecular simulation, RNA *in situ* hybridization, RNA sequencing, visual opsin, salmonid

## Abstract

Salmonids are ideal models as many species follow a distinct developmental program from demersal eggs and a large yolk sac to hatching at an advanced developmental stage. Further, these economically important teleosts inhabit both marine- and freshwaters and experience diverse light environments during their life histories. At a genome level, salmonids have undergone a salmonid-specific fourth whole genome duplication event (Ss4R) compared to other teleosts that are already more genetically diverse compared to many non-teleost vertebrates. Thus, salmonids display phenotypically plastic visual systems that appear to be closely related to their anadromous migration patterns. This is most likely due to a complex interplay between their larger, more gene-rich genomes and broad spectrally enriched habitats; however, the molecular basis and functional consequences for such diversity is not fully understood. This study used advances in genome sequencing to identify the repertoire and genome organization of visual opsin genes (those primarily expressed in retinal photoreceptors) from six different salmonids [Atlantic salmon (*Salmo salar*), brown trout (*Salmo trutta*), Chinook salmon (*Oncorhynchus tshawytcha*), coho salmon (*Oncorhynchus kisutch*), rainbow trout (*Oncorhynchus mykiss*), and sockeye salmon (*Oncorhynchus nerka*)] compared to the northern pike (*Esox lucius*), a closely related non-salmonid species. Results identified multiple orthologues for all five visual opsin classes, except for presence of a single short-wavelength-sensitive-2 opsin gene. Several visual opsin genes were not retained after the Ss4R duplication event, which is consistent with the concept of salmonid rediploidization. Developmentally, transcriptomic analyzes of Atlantic salmon revealed differential expression within each opsin class, with two of the long-wavelength-sensitive opsins not being expressed before first feeding. Also, early opsin expression in the retina was located centrally, expanding dorsally and ventrally as eye development progressed, with rod opsin being the dominant visual opsin post-hatching. Modeling by spectral tuning analysis and atomistic molecular simulation, predicted the greatest variation in the spectral peak of absorbance to be within the Rh2 class, with a ∼40 nm difference in λ_*max*_ values between the four medium-wavelength-sensitive photopigments. Overall, it appears that opsin duplication and expression, and their respective spectral tuning profiles, evolved to maximize specialist color vision throughout an anadromous lifecycle, with some visual opsin genes being lost to tailor marine-based vision.

## Introduction

Salmonids are a group of closely related teleost species of high economical value with an anadromous lifestyle, inhabiting both freshwater and marine environments as part of their life cycle. These habitats include lakes, rivers, and marine ecosystems from coastal areas to open ocean, all with very different optical characteristics. In the water column, the downwelling light environment changes rapidly with depth as photons are absorbed and scattered by water molecules and components such as dissolved organic matter, chlorophyll in phytoplankton and suspended soil. The concentration of these components varies in fresh and marine waters causing local variations in spectral irradiance (Jerlov, 1976; Levine and MacNichol, 1982; Partridge and Cummings, 1999). In general, the light of shorter and longer wavelengths are limited to the upper water column, while short-wavelengths in the blue part of the light spectrum (around 480 nm) penetrates the deepest (Levine and MacNichol, 1982; Douglas and Djamgoz, 1990). As such, constantly changing lighting conditions (i.e., diurnal variation in both the spectrum and intensity of light environment), place great selective pressure on the visual system of many fishes, especially those that dwell in rivers or near the surface of estuaries and/or oceans, where visual capabilities are likely to change during development, as well as in response to daily and annual fluctuations of light (Evans and Browman, 2004; Carleton et al., 2020).

Visual photoreception is fundamentally dependent on photopigments, which consist of an opsin protein bound to a light-sensitive chromophore, located in the outer membrane of ocular rods and cones (Terakita, 2005). Being sensitive to different wavelengths of light, due in part to the opsin protein sequence present (Carleton et al., 2020), visual photopigments directly translate environmental light information (photons) in the first step of the phototransduction cascade that ultimately leads to a perceived “colored” image of the external world that is generated in the visual centers of the brain. In vertebrates, the cone visual opsins used for color vision are generally divided into four separate classes based on distinct spectral sensitivities with peak absorbance (i.e., λ_*max*_) values within the ultraviolet (UV) (SWS1, λ_*max*_ = 355–445 nm), blue (SWS2, λ_*max*_ = 400–470 nm), green (RH2, λ_*max*_ = 460–530 nm) and red (LWS, λ_*max*_ = 500–575 nm) regions of the light spectrum (Davies et al., 2012). While cones require bright light (i.e., for photopic vision) to function, rods containing RH1 (λ_*max*_ = 460–530 nm, but is typically ∼500 nm for most shallow water or terrestrial species) are used for dim light or scotopic vision (Yokoyama, 2000).

In general, the photoreceptive or visual possibilities of a species is dependent on the opsin genes existing in the genome and specific spatial and temporal expression patterns. The teleost visual system is particularly diverse among vertebrates, where tandem duplications and whole genome duplications have been characteristically important in shaping the evolution and function of spectrally distinct photoreceptive pathways (Kuraku et al., 2008; Rennison et al., 2012). In the common ancestor of all salmonids, a salmonid-specific fourth whole genome duplication, Ss4R, occurred approximately ∼80 million years ago (mya) (Allendorf and Thorgaard, 1984; Lien et al., 2016). However, genomic analyzes of visual opsins in Atlantic salmon and rainbow trout have revealed that many opsin genes have been lost (Lin et al., 2017; Musilova et al., 2019). For those opsin genes still retained in the genome, it is likely that they have functionally evolved in response to selective pressures such as the external light environment to regulate their expression profiles and/or encode photopigments that exhibit spectrally distinct characteristics *via* changes in amino acids at key tuning sites that spectrally tune these photopigments to different wavelengths (Yokoyama, 2000). In recent years, an increasing number of fish genomes have been sequenced, highlighting the importance of habitat diversity in aqueous environments as a driving force in fish opsin gene evolution (Lin et al., 2017). Based on studies in agnathans, it has been proposed, for many vertebrate classes including teleosts (Davies et al., 2012), that alterations in gene copies and subsequent mutations play an important role in the evolution of color vision and that spectral tuning of visual photopigments often strongly reflects ecology, especially regarding the light environment (Davies et al., 2009).

Unique features of the teleost eye include continued retinal growth and plasticity throughout postembryonic changes and development (Evans and Fernald, 1990). Many marine species, such as Atlantic cod, follow an indirect developmental program, where they hatch with poorly differentiated retinas followed by a long larval period with a pure-cone retina (Balon, 1985). The rods develop during metamorphosis (i.e., the transition to the juvenile stage), supported by a large increase in the expression of rod opsin (Valen et al., 2016). Conversely, salmonids follow a more direct developmental program, with demersal eggs and a large yolk sac, where they hatch at an advanced developmental stage (Kendall et al., 1984). Studies in salmonids show that expression of visual opsins occurs before hatching and that cones expressing *sws1* (“UV”), *rh2* (“green”), and *lws* (“red”), as well as rod opsin, appear embryonically, while cones expressing *sws2* appear after hatching (Cheng et al., 2007). Further, phenotypic plasticity in the visual system of salmonids related to sea-river migration has been shown by temporal loss and gain of UV cones containing Sws1 photopigments (Allison et al., 2006), as well as a shift from UV to blue sensitivity during salmonid retinal development that is related to a change in lifestyle from rivers with abundant UV light to deeper oceanic waters (Cheng and Flamarique, 2007).

Utilizing recent advances in genome sequencing that provided access to the whole genomes of several salmonids (Macqueen et al., 2017), this study identified the complete complement of visual opsin genes in six salmonids and compared their phylogenetic diversity and rediploidization to that of the northern pike, a sister linage that did not undergo Ss4R (i.e., the salmonid-specific whole genome duplication event) (Rondeau et al., 2014). Developmental opsin expression patterns were monitored, showing differential temporal expression within specific opsin classes. Further, by using a combination of spectral tuning site interrogation and atomistic molecular simulation analyzes, spectral profiles for each Atlantic salmon visual photopigment (i.e., Sws2, Rh2, and Lws classes) were determined and functional consequences discussed within the context of teleost development and visual ecology.

## Materials and Methods

### Animals

Eggs and sperm from Atlantic salmon (*Salmo salar*) were obtained from MOWI, Tveitevågen, Norway. Fertilization took place at an approved laboratory facility at High Technology Center, University of Bergen, Norway, where alevins were raised until the stages, after hatching at 555 day degrees (dd) and just before first feeding at 720 dd. These stages correspond approximately to relative ages 310 and 390 in Gorodilov (1996). The “dd” nomenclature refers to the average temperature per day (6° with a standard deviation of 0.5) multiplied by the number of days. All experiments described followed local animal care guidelines and were given ethical approval by the Norwegian Food Safety Authority. The study complied with ARRIVE guidelines (Percie du Sert et al., 2020).

### Molecular Cloning of Visual Opsins

Eyes were collected from Atlantic salmon alevins at the stage just before first feeding, where total RNA was isolated by TRI reagent (Sigma, St. Louis, MO, United States), then treated with DNase I by using a TURBO DNA-free™ Kit (ThermoScientific, Waltham, MA, United States). Complementary DNA (cDNA) was reversely transcribed by using a SuperScript III kit (Invitrogen, Carlsbad, CA, United States) as recommended by the manufacturer’s instructions. Initial *in silico* analyzes of the Atlantic salmon genome database ICSASG_v2 (Lien et al., 2016) was conducted by using BLASTP or BLASTN *via* NCBI (Sayers et al., 2019) with zebrafish (*Danio rerio*) visual opsin sequences being used as bait for gene mining. To generate full-length visual opsins, primers were designed that anneal to published or predicted untranslated regions (UTRs) or to regions containing start/stop codons. An Advantage^®^2 PCR Kit (TaKaRa, Japan) was used to perform amplification reactions by applying PCR or nested PCR approaches (see Supplementary Table 1) over 35 cycles. PCR products were extracted from agarose gels using a MinElute^®^Gel Extraction Kit (Qiagen, Germany), before being cloned into StrataClone PCR Cloning vector pSC-A-amp/kan (Agilent Technologies, LA Jolla, CA, United States). Correct clones were sequenced at the University of Bergen Sequencing Facility, with nucleotide sequences being deposited to the GenBank database with Accession Numbers (ON456431-ON456441).

### Sequence and Phylogenetic Analyzes

*In silico* search in salmonid genome databases [brown trout (*Salmo trutta*), Chinook salmon (*Oncorhynchus tshawytcha*), coho salmon (*Oncorhynchus kisutch*), rainbow trout (*Oncorhynchus mykiss*), sockeye salmon (*Oncorhynchus nerka*)] and in the northern pike (*Esox lucius*) was performed to identify the visual opsins of the respective species. The search was performed by BLASTP or BLASTN on NCBI (Sayers et al., 2019) or Ensembl (Cunningham et al., 2018) using the Atlantic salmon visual opsins in the search. In addition, the sequences used for Atlantic salmon *rh1-2.1* and *rh1-2.2* are from Lin et al. (2017). The coding sequences of annotated or putative opsin genes in the genomes were aligned to the Atlantic salmon visual opsins using Vector NTI9 software (Invitrogen, Carlsbad, CA, United States) and ClustalX2 (Larkin et al., 2007) to evaluate the coding sequence. The genomes of Atlantic salmon, brown trout, Chinook salmon, rainbow trout, coho salmon, and an updated version of northern pike were published on Ensembl (version 104), and recently updated versions of Atlantic salmon and rainbow trout have been published (version 106). The most recent sequences from NCBI and Ensembl have been used in the evaluation of open reading frame (ORF). Sequences determined to yield intact ORFs, cloned sequence for Atlantic salmon and either NCBI or Ensembl sequence for the other species, were used in the phylogenetic analysis, whereas partial sequences and/or sequences derived from pseudogenes were omitted. A codon-matched nucleotide sequence alignment of 90 ray-finned fish opsin coding regions was generated by ClustalW (Higgins et al., 1996) and manually manipulated to refine the accuracy of cross-species comparison. Specifically, the alignment incorporated opsin coding sequences of visual photopigments identified in the genomes of several salmonid species and a phylogenetically related species (i.e., *E. lucius*, northern pike) compared to orthologous visual opsin sequences expressed in the retina of *D. rerio* (zebrafish). All five visual opsin classes (i.e., *lws*, *sws1*, *sws2*, *rh2*, and *rh1*) were included, with zebrafish vertebrate ancient (va) opsin sequences (*va1* and *va2*) used collectively as an outgroup given that this opsin type is a sister clade to all five visual photopigment classes. Phylogenetic analyses of 1,000 replicates were conducted in MEGA11 (Tamura et al., 2021), with evolutionary histories being inferred by using the Maximum Likelihood method and General Time Reversible model (Nei and Kumar, 2000). The percentage of trees in which the associated taxa clustered together is shown next to the branches. Initial trees for the heuristic search were obtained by applying Neighbor-Joining and BioNJ algorithms (Saitou and Nei, 1987) to a matrix of pairwise distances estimated using the Maximum Composite Likelihood (MCL) approach (Tamura and Nei, 1993). The tree was drawn to scale, with branch lengths measured in the number of substitutions per site. A total of 996 positions was present in the final dataset, with all positions with less than 95% site coverage being eliminated. That is, fewer than 5% alignment gaps, missing data, and ambiguous bases were allowed at any position.

### Prediction of λ_*max*_ Values of Visual Photopigments Expressed in the Eyes of Atlantic Salmon

Currently, it is known that λ_*max*_ values of vertebrate photopigments are influenced by ∼40 known amino acid tuning sites (Davies et al., 2012; Musilova et al., 2019). Using conventional bovine rod opsin (Accession Number NP001014890) numbering, spectral peak of absorbance values were reliably predicted for Sws1, Rh1, and Lws photopigments using manual interrogation as outlined in Musilova et al. (2019). Particular attention was made at site 86, which is important for UV-sensitivity (UVS) in Sws1 photopigments (Cowing et al., 2002). For Rh1 photopigments, sites 83, 90, 96, 102, 113, 118, 122, 124, 132, 164, 183, 194, 195, 207, 208, 211, 214, 253, 261, 265, 269, 289, 292, 295, 299, 300, and 317 were analyzed as being spectrally important (Sakmar et al., 1991; Chan et al., 1992; Hope et al., 1997; Yokoyama et al., 1999, 2007, 2008; Yokoyama, 2000, 2008; Hunt et al., 2001; Janz and Farrens, 2001; Davies et al., 2007, 2012; Kuraku et al., 2008). Similarly, spectral tuning sites 164, 181, 261, 269, and 292 were investigated to calculate Lws photopigment λ_*max*_ values (Yokoyama, 2000; Davies et al., 2012). To determine the λ_*max*_ values of four Rh2 and an Sws2 visual photopigment, molecular dynamics simulations-based modeling approaches were applied (Patel et al., 2018, 2020). In this approach, molecular dynamics simulations were carried out using 3D homology models of vertebrate Rh2 and Sws2-type visual photopigments for which λ_*max*_ values have been experimentally measured. Molecular simulations were then used to develop simple statistical models using parameters describing conformation and fluctuation of the 11-*cis* retinal chromophore and attached lysine residue, which predicted λ_*max*_ values with high accuracy. Once determined, representative dark spectra for all visual photopigments were generated using a standard A_1_-based rhodopsin template (Govardovskii et al., 2000).

### RNA Sequencing Analyzes of Atlantic Salmon Sampled During Development

RNA sequencing reads of the developmental series (whole embryos and alevins) were obtained by downloading from SRA on NCBI (Sayers et al., 2019) (BioProject PRJNA72713, BioSample SAMN02864156-SAMN02864171) associated with the publication of the Atlantic salmon genome (Lien et al., 2016). The developmental stage, day degrees (dd), was identified by calculating the number of days associated with the BioSamples and the incubation temperature (9.4°C) (von Schalburg et al., 2014). The samples were aligned to the published Atlantic salmon genome (GCF_000233375.1) using Bowtie2 (Langmead and Salzberg, 2012), read counts were generated using Samtools (Li et al., 2009) and the reads where normalized to the sample with the lowest number of reads. A heatmap presented by the logfold2 change of individual visual opsin genes was made by pheatmap (Kolde, 2019).

### Riboprobes and Localization of Visual Opsin Transcripts by RNA *in situ* Hybridization

Preparation of digoxigenin DIG-labeled riboprobes for the visual opsins of Atlantic salmon were prepared following the manufacturer’s instructions (Roche Diagnostics, Germany). In the synthesis of riboprobes, specific PCR products were used as template for the reaction (Thisse and Thisse, 2008), where synthesized probes were precipitated by LiCl and ethanol together with tRNA (Roche Diagnostics, Germany). Nucleotide sequence alignments showed that sequence identity between visual opsin targets was between 50 and 65%, thus ensuring no cross-hybridization between opsin classes. For the four *rh2* genes, the identity in the coding region between the four genes is ∼87% and cross-hybridization was likely to occur. Therefore, the overall Rh2 class expression was analyzed using all four *rh2* as templates in the probe synthesis reaction to generate a probe mix of all genes. For the four *lws* genes, the probe targets the coding region with an overall sequence identity of ∼87%. As such, both *lws*2 and *lws*3 were used as template, however, the probe hybridized to all *lws* genes as the individual probes have a sequence similarity above 90% to paralogues not used during the generation of the probe by PCR. Alevins (555 and 720 dd, hatched Atlantic salmon larvae living on their yolk sac) were euthanized with an overdose of tricaine (MS-222, Sigma, United States) before fixation in 4% paraformaldehyde-buffered PBS and further processed as described in Eilertsen et al. (2014). Cryosectioning of consecutive frontal sections (10 μm) was performed on a Leica CM 3050S cryostat (Leica Biosystems, Germany) and before storage at −20°C, the tissues were air dried for 1 h at room temperature and then incubated for 30 min at 65°C. RNA *in situ* hybridization was carried out as described by Sandbakken et al. (2012). Images were taken with a Leica DFC 320 digital camera (Leica Microsystems, Germany) attached to a Leica DM 6000B microscope (Leica Microsystems, Germany). Adobe Photoshop CC (San Jose, CA, United States) was used to adjust image brightness and contrast, as well as for displaying schematic overviews of visual opsin expression patterns at different developmental stages.

## Results

### Identification of Visual Opsins in Atlantic Salmon

*In silico* gene mining of the Atlantic salmon genome and reviewing the literature allowed for the identification of *rh1-1, rh1-2.1, rh1-2.2*, *sws1-1*, *sws1-2*, *sws2*, *rh2-1*, *rh2-2*, *rh2-3*, *rh2-4*, *lws1*, *lws2*, *lws3*, and *lws4* visual opsin genes (Table 1, Supplementary Table 2, and Supplementary Figures 1–7). However, *rh1-2.1, rh1-2.2*, and *sws1-2* were evaluated to be pseudogenes by Lin et al. (2017) and this present study (Supplementary Figures 2, 6, 7). An evaluation of *sws1-2* showed a 6 base pair (bp) deletion and a premature stop codon, indicating that Sws1-2, if translated, would be truncated. The results of this study show that an additional *lws* gene exists in the salmon genome, here named *lws4*, when compared to the findings of Lin et al. (2017), as indicated in Musilova et al. (2019). Using molecular cloning, the sequence of 11 full-length visual opsins were identified to be expressed in Atlantic salmon.

**TABLE 1:** Visual opsin repertoires in six salmonids and northern pike.

Species	*rh1*	*sws1*	*sws2*	*rh2*	*lws*	Not included in the phylogenetic analyses
Atlantic salmon (*Salmo salar*)	3	2	1	4	4	*rh1-2.1*, *rh1-2.2*, *sws1-2*
Brown trout (*Salmo trutta*)	2	2	1	4	4	*rh1-2.1*, *rh1-2.2*, *sws1-2*
Chinook salmon (*Oncorhynchus tshawytcha*)	2	2	1	4	4	*rh1-2.1*, *lws3*
Coho salmon (*Oncorhynchus kisutch*)	2	2	1	5	4	*rh1-2.1*, *lws3*
Northern pike (*Esox lucius*)	1	1	0	4	4	
Rainbow trout (*Oncorhynchus mykiss*)	2	2	1	5	4	*rh2-1.2*, *lws3*, *lws4.2*
Sockeye salmon (*Oncorhynchus nerka*)	1	2	1	5	3	*lws4*

### Identification of Visual Opsins in Other Salmonids and Northern Pike

Using *in silico* analyzes, visual opsins were identified in six additional salmonids as listed above, as well as in the northern pike, a member of the closest related diploid sister-group to salmonids (Rondeau et al., 2014; Table 1 and Supplementary Table 2). In brown trout, *rh1-1*, *rh1-2.1, rh1-2.2*, *sws1-1*, *sws1-2*, *sws2*, *rh2-1*, *rh2-2*, *rh2-3*, *rh2-4*, *lws1*, *lws2*, *lws3*, and *lws4* were identified. Closer inspection showed that *rh1.2.1* and *rh1.2.2* are pseudogenes as it possesses several indels within the ORF, which is consistent with the same gene being a pseudogene in Atlantic salmon (Supplementary Figure 6). In addition, *sws1-2* had the same 6 bp deletion as present in Atlantic salmon *sws1-2*, as well as additional indels in the ORF (Supplementary Figure 7). As such, these genes were not included in the phylogenetic analyzes. The following genes were identified in Chinook salmon: *rh1-1*, *rh1-2.1*, *sws1-1*, *sws1-2*, *sws2*, *rh2-1*, *rh2-2*, *rh2-3*, *rh2orf-4*, *lws1*, *lws2*, *lws3*, and *lws4*; however, both *lws3* (partial sequence) and *rh1-2.1* contained several indels (Supplementary Figure 6) and were not included in the phylogenetic analyzes. In coho salmon, *rh1-1*, *rh1-2.1*, *sws1-1*, *sws1-2*, *sws2*, *rh2-1.1*, *rh2-1.2*, *rh2-2*, *rh2-3*, *rh2-4*, *lws1*, *lws2*, *lws3*, and *lws4* were identified, revealing an additional *rh2* when compared to Atlantic salmon. Closer inspection showed that the ORF of *rh1-2.1* had several indels and is, therefore, a pseudogene as in Atlantic salmon (Supplementary Figure 6). The *lws3* gene was not included in the phylogenetic analyzes as it lacks sequence at the 5′ end and contains several indels. In rainbow trout, *rh1-1*, *rh1-2.2*, *sws1-1*, *sws1-2*, *sws2*, *rh2-1.1*, *rh2-1.2*, *rh2-2*, *rh2-3*, *rh2-4*, *lws1*, *lws2*, *lws3*, *lws4*, and *lws4.2* genes were found. As in Atlantic salmon, rainbow trout *rh1-2.2* contained several indels and has become pseudogenized (Supplementary Figure 6). In this study, an extra *rh2-1.1* gene was identified compared to Lin et al. (2017) but the *lws*3 gene was evaluated to be a pseudogene, as in Lin et al. (2017), with several indels and a truncated 3′ end. At Ensembl version 106, an extra *lws4* was identified at chromosome 15 (named *lws4.2*). It has a 33 bp deletion in the third transmembrane region and were consider as not functional. In the sockeye salmon genome, the following visual opsin genes were identified: *rh1-1*, *sws1-1*, *sws1-2*, *sws2*, *rh2-1.1*, *rh2-1.2*, *rh2-2*, *rh2-3*, *rh2-4*, *lws1*, *lws2*, and *lws4.* As in coho salmon and rainbow trout, an additional *rh2* gene was also identified. The sockeye salmon genome is currently only hosted by Ensembl Rapid Release, and the search for opsins was done at NCBI, and a search for *rh1-2.1* and *rh1-2.2* genes contained within NCBI databases failed to yield any hits. The *lws4* gene was shown to possess an indel in the ORF and was not included in the phylogenetic analyzes. As revealed in Lin et al. (2017), the northern pike genome contains *rh1-1*, *sws1-1*, *rh2-1*, *rh2-2*, *rh2-3*, *rh2-4*, *lws1*, *lws2*, *lws3*, and *lws4*, and in the updated pike genome at Ensembl database (version 104, Eluc_v4 assembly) a fourth *rh2-4* is included.

### Phylogenetic Analyzes of Visual Opsins

**FIGURE 1:**
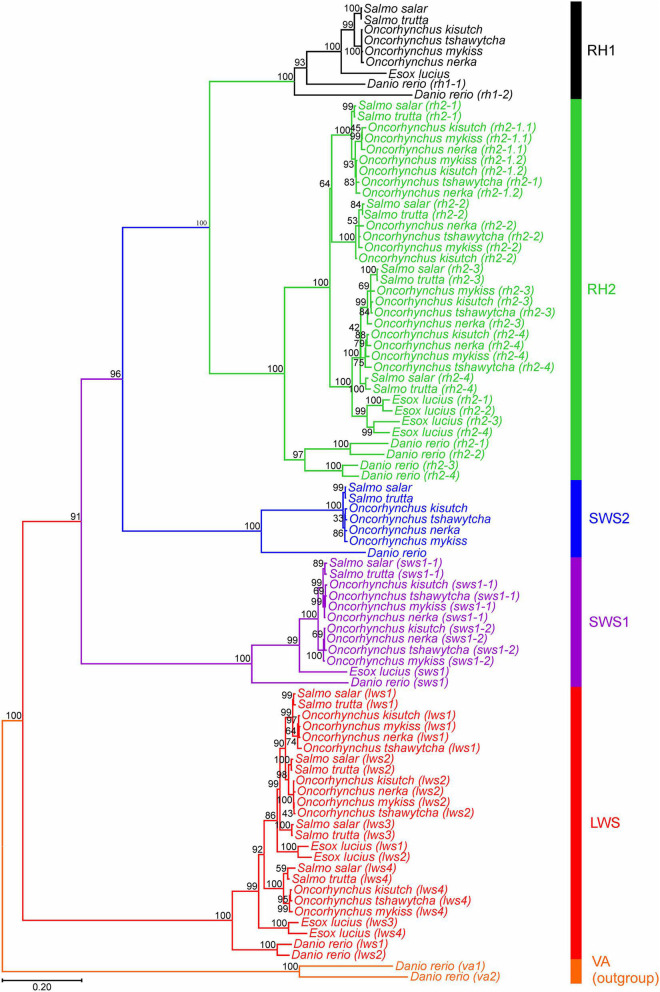
Opsin evolutionary history as inferred by using the Maximum Likelihood method and General Time Reversible model (Nei and Kumar, 2000). The tree with the highest log likelihood (–21,458.16) is shown. The percentage of trees for which the associated taxa clustered together is shown next to the branches. Initial tree(s) for the heuristic search were obtained automatically by applying Neighbor-Join and BioNJ algorithms (Saitou and Nei, 1987), with a bootstrap value of 1,000, to a matrix of pairwise distances estimated using the Maximum Composite Likelihood (MCL) approach (Tamura and Nei, 1993), and then selecting the topology with superior log likelihood value. A discrete Gamma distribution was used to model evolutionary rate differences among sites [5 categories (+*G*, parameter = 1.0379)]. The rate variation model allowed for some sites to be evolutionarily invariable [(+*I*), 8.84% sites]. The tree is drawn to scale, with branch lengths measured in the number of nucleotide substitutions per site (indicated by the scale bar). This analysis involved 90 nucleotide sequences, where photopigment genes from six salmonid species and northern pike across five visual opsin classes (*lws*, *sws1*, *sws2*, *rh2*, and *rh1*) were compared. *Danio rerio* (zebrafish) vertebrate ancient (va) opsin 1 and 2 (*va1* and *va2*) sequences were used as an outgroup. Codon positions included were 1st + 2nd + 3rd + noncoding. All positions with less than 95% site coverage were eliminated, i.e., fewer than 5% alignment gaps, missing data, and ambiguous bases were allowed at any position (partial deletion option). There was a total of 996 positions in the final dataset. Evolutionary analyses were conducted in MEGA11 (Tamura et al., 2021).

A maximum likelihood tree based on a codon-matched alignment of nucleotide sequences was generated to show the putative evolutionary relationship between differing salmonid visual opsin gene copy numbers and related visual opsin genes of the northern pike. Specifically, Figure 1 shows the presence of several *lws*, *sws1, sws2*, and *rh2* cone opsin genes, with all species having one functional *rh1* gene. All salmonids have an additional *sws1* gene compared to northern pike (Table 2); however, the second *sws1-2* sequence was only included in the phylogenetic analyzes for Chinook salmon, coho salmon, rainbow trout, and sockeye salmon since these genes are intact and likely to be functional photopigments. As shown in Lin et al. (2017), northern pike lack a *sws2* gene in the genome, while salmonids all possess one intact *sws2* gene. The *lws1* and *lws2* genes were present for all salmonids and northern pike, while the *lws3* gene was only included for Atlantic salmon, brown trout and northern pike since it was not identified, present as a partial sequence or assigned as a pseudogene in other salmonids. The *lws4* gene was not identified in sockeye salmon but were present for the other species. The maximum likelihood tree of the *rh2* opsin class revealed that salmonids possess 3 to 5 *rh2* genes: coho salmon, rainbow trout, and sockeye salmon all have five *rh2* genes, with phylogenetic analyzes showing that there has been an extra duplication of the *rh2-1* gene in these species. The *rh2* opsin genes in salmonids are tandemly duplicated in a gene array where each gene is located in close proximity to each other on the same chromosome for a particular species (Supplementary Table 2). Phylogenetically, *rh2-1/rh2-2* and *rh2-3/rh2-4* appear to branch together.

**TABLE 2:** Area under the RMSF(_*LYS*+*RET*_) curve and median values of various angles used to predict λ_*max*_ values for each of the four Atlantic salmon Rh2 photopigments and a single Sws2 visual photopigment using the statistical models.

	Angle 3	Torsion 3	Torsion 12	Predicted λ_*max*_
Sws2	132.33	2.14	−5.1	420.04

	**Torsion 15**	**RMSF(_*LYS*__+__*RET*_)**		**Predicted λ_*max*_**

Rh2-1	3.64	0.79		471.5
Rh2-2	3.06	0.76		475.5
Rh2-3	0.41	1.12		511.2
Rh2-4	0.84	1.1		506.4

### Predicted λ_*max*_ Values of Visual Photopigments in Atlantic Salmon

**FIGURE 2:**
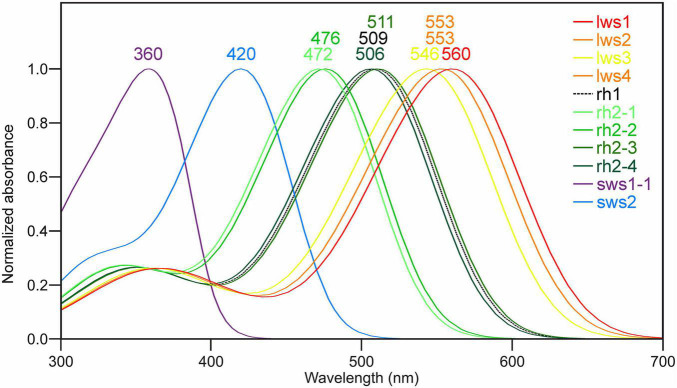
Predicted spectral peaks of absorbance for Atlantic salmon visual photopigments. The schematic shows predicted λ_*max*_ values using normalized A_1_-based rhodopsin templates (Govardovskii et al., 2000) as follows: Sws1 = 360 nm; Sws2 = 420 nm; Rh2-1 = 472 nm; Rh2-2 = 476 nm; Rh2-3 = 511 nm; Rh2-4 = 506 nm; Lws1 = 560 nm; Lws2 = 553 nm; Lws3 = 546 nm; Lws4 = 553 nm, as well as Rh1 = 509 nm.

For Lws, Sws1, and Rh1 photopigments, it is possible to accurately determine spectral peak of absorbance (λ_*max*_) values using manual interrogation (e.g., Davies et al., 2012; Musilova et al., 2019). Specifically, analyses of the ∼40 known amino acid tuning sites showed that Sws1 was predicted to have a λ_*max*_ value at 360 nm due to the presence of Phe86, (Cowing et al., 2002), whereas the spectral peak of Atlantic salmon Rh1 was determined to be 509 nm (Figure 2 and Table 2), assuming the use of a vitamin A_1_-based retinal chromophore in both cases. Similar inspection of the four Lws photopigments in Atlantic salmon predicted a 14 nm span in their respective λ_*max*_ values based on the presence of spectral tuning sites 164, 181, 261, 269, and 292 (Yokoyama, 2000; Davies et al., 2012). Specifically, these spectral peaks ranged from 546 nm (for Lws3) to 560 nm (for Lws1), whereas the λ_*max*_ value was predicted to be 553 nm for both Lws2 and Lws4 photopigments (Figure 2).

**FIGURE 3:**
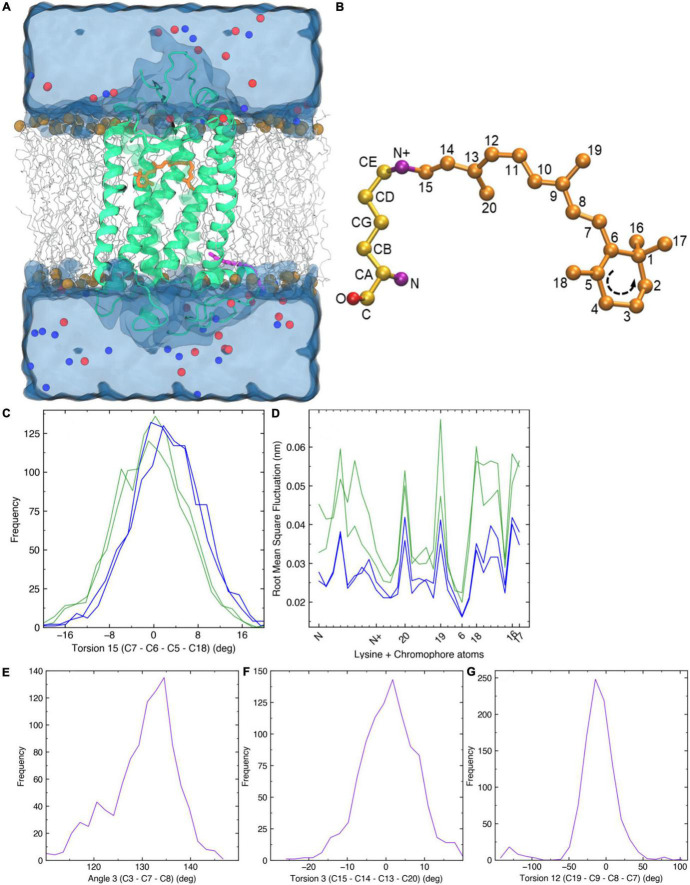
Molecular dynamics simulations. **(A)** A 3D homology structure of Atlantic salmon Rh2-1 cone opsin (green) with the chromophore (orange) bound covalently to K296 of the opsin protein. It is embedded in a phospholipid bilayer (gray, carbon atoms; brown, phosphorus atoms) and surrounded by water molecules (blue). Blue and red spheres indicate positive and negative counter ions, respectively. **(B)** 11-*cis* retinal attached to a lysine residue *via* a Schiff base linkage. **(C)** Frequency distribution of C7–C6–C5–C18 torsion angle 15 (T15) observed in each Rh2 visual photopigment simulation. Blue and green colors indicate spectral sensitivities of each visual photopigment. **(D)** Root mean square fluctuation of 11-*cis* retinal attached to a lysine residue (LYS + RET). Horizontal axis represents atoms along the LYS + RET (see panel **B**). Sequence of β-ionone ring is indicated in **B**. **(E,G)** Frequency distribution of C3–C7–C8 geometric angle 3 (A3), C15–C14–C13–C20 torsion angle 3 (T3), and C19–C9–C8–C7 torsion angle 12 (T12) observed in a Sws2 visual photopigment simulation. Purple color indicates spectral sensitivity of visual photopigments.

As λ_*max*_ values for Sws2 and Rh2 photopigment classes are difficult to accurately predict from amino acid sequences alone, molecular dynamics simulations-based modeling approaches were applied (Patel et al., 2018, 2020). A two-term statistical model, 475.628 + (−8.720 ×Torsion 15) + (34.925 ×RMSF_(LYS__+__*RET)*_), predicted the λ_*max*_ values of four Rh2 cone visual photopigments expressed in Atlantic salmon (Figure 3). In this model, Torsion 15 was a median value of the dihedral angle formed by C7–C6–C5–C18 atoms of 11-*cis* retinal (Figure 3C) and RMSF_(LYS__+__*RET)*_ was a value of area under the root mean square fluctuation curve of the chromophore bound to lysine residue in the chromophore binding site (Figure 3D; Patel et al., 2018). Conversely, the λ_*max*_ value of Atlantic salmon Sws2 cone visual photopigment was predicted by a three-term model: 2677.5348 − (−17.052 ×Angle 3) + (5.1634 ×Torsion 3) + (2.3642 ×Torsion 12), where Angle 3 (C3–C7–C8), Torsion 3 (C15–C14–C13–C20), and Torsion 12 (C19–C9–C8–C7) were the median values obtained from molecular dynamics simulations (Figures 3E–G; Patel et al., 2020). To do this, molecular dynamics simulations using each visual photopigment homology model were performed as previously described (Patel et al., 2018, 2020). Specifically, 100 ns long molecular dynamics simulation for all five visual photopigment systems were performed using a GROMACS simulation package (Van Der Spoel et al., 2005). RMSF and median values of the angles were then calculated from each 100 ns simulation and these values were applied to the statistical models to predict all λ_*max*_ values. Modeling results showed that Sws2 had a λ_*max*_ value at 420 nm, whereas the predicted λ_*max*_ values of Atlantic salmon Rh2 photopigments span a range of almost 40 nm, from 472 to 511 nm, where Rh2-1/Rh2-2 have λ_*max*_ values around 475 nm and Rh2-3/Rh2-4 around 510 nm (Figure 2 and Table 2).

### Differential Expression of Visual Opsins in Atlantic Salmon Development

**FIGURE 4:**
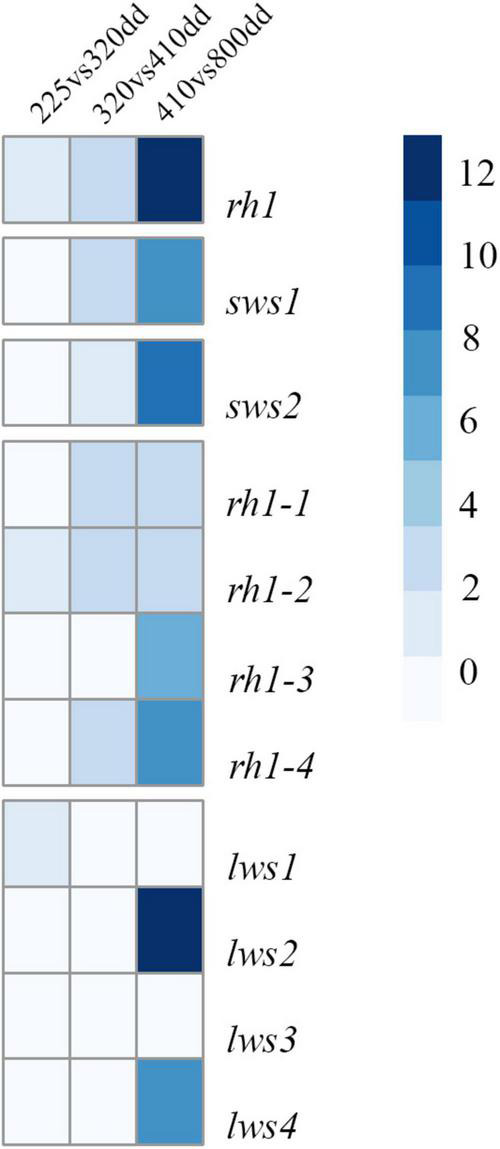
A heatmap of expression levels for individual visual opsin genes in Atlantic salmon. The expression levels identified by RNA sequencing of whole embryos and alevins is presented by logfold2 change. The heatmap shows expression changes observed during development, revealing a significant shift in expression levels from 410 to 800 dd.

The relative expression profiles of visual opsins in whole embryos and alevins are shown in Figure 4, where the developmental series ranges from the eye pigmentation stage to first feeding. In addition, Table 3 shows the mean relative level of expression by normalized counts at each developmental stage, where reads were normalized to the sample with the lowest number of reads (Supplementary Table 3 shows the individual normalized counts of the developmental series). The results reveal that the expression of visual opsins is substantial at 800 dd, corresponding to the developmental stage around first feeding. However, already at 410 dd, before hatching, opsin expression levels increased within the *rh2* and *rh1-1* classes. At 800 dd, rod opsin was at a maximal expression level (Table 3). Among the four *rh2* opsin genes, *rh2-3* and *rh2-4* were expressed at higher levels at all stages when compared to *rh2-1* and *rh2-2*. While *lws2* had the highest expression level within the *lws* opsin class, both *lws1* and *lws3* exhibited little or no expression at embryo and alevin stages. Overall, changes in expression levels during development (Figure 4) revealed that the largest shift in transcript levels occurred between 410 and 800 dd.

**TABLE 3:** The mean of normalized counts showing the expression of visual opsins in Atlantic salmon in a developmental series.

	225 dd	320 dd	410 dd	800 dd
*rh1-1*	1	1	10	30,740
*sws1-1*	0	1	3	698
*sws2*	0	0	1	351
*rh2-1*	9	4	20	211
*rh2-2*	3	6	22	219
*rh2-3*	0	0	10	754
*rh2-4*	0	1	4	513
*lws1*	0	1	0	2
*lws2*	0	0	0	1,240
*lws3*	1	0	0	8
*lws4*	0	0	2	304

### Expression of Visual Opsins in the Developing Eye

**FIGURE 5:**
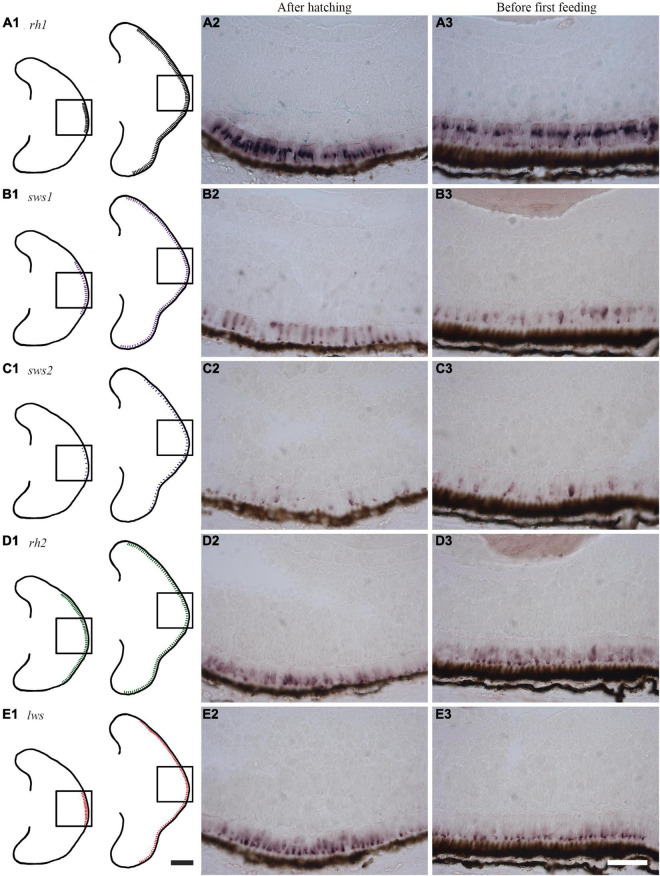
Expression of visual opsins in the developing Atlantic salmon eye. **(A1–E1)** Schematics showing the expression patterns of visual opsins after hatching (555 dd) and before first feeding (720 dd) as obtained by RNA *in situ* hybridization. **(A1–A3)** After hatching, *rh1* is strongly expressed in rod photoreceptor cells in the central retina, with an expression pattern that spreads dorsally and ventrally before first feeding. **(B1–B3)** The single cone opsin *sws1* is expressed in a broader region of the central retina compared to *rh1* at hatching: this expression spreads dorsally and ventrally before first feeding. **(C1–C3)** After hatching, the single cone opsin *sws2* has scattered expression in the central retina. Before first feeding, *sws2*-positive cells are sparse, but they subsequently spread dorsally and ventrally. **(D1–D3)** Probes detecting all four *rh2* genes expressed in double cones reveal that *rh2-*positive cells are the most widely distributed after hatching. Before first feeding, expression levels are highest close to the retinal pigment epithelium (RPE), before spreading dorsally and ventrally. **(E1–E3)** Probes detecting all four *lws genes* (again in double cones) show a central distribution of *lws* before hatching. When approaching first feeding, *lws* expression spreads dorsally and ventrally with expression that is located towards the RPE. Scale bars, 200 μm for schematic drawings and 50 μm for histological sections.

The retinal expression patterns of visual opsins in Atlantic salmon after hatching and just before first feeding (555 and 720 dd) were revealed by RNA *in situ* hybridization using opsin class-specific riboprobes (Figure 5 and Supplementary Figure 8). Strong expression of rod opsin (*rh1*) was detected in the central retina after hatching, with expression that spread dorsally and ventrally as development progressed (Figures 5A1–A3). Both *sws1* (Figures 5B1–B3) and *sws2* (Figures 5C1–C3) were also expressed centrally after hatching, with opsin-positive cones spreading dorsally and ventrally in a similar manner to that of *rh1*; however, the number of *sws2*-positive cones were sparse when compared to those expressing *sws1*, especially after hatching. The expression of the four *rh2* genes after hatching was more widespread than the other visual opsin genes, even though *rh2* expression was detected in the central retina (Figures 5D1–D3). Before first feeding, *rh2* opsins were also expressed dorsally and ventrally (Figures 5D1–D3). For the Lws class, opsin expression was initially located centrally, spreading dorsally, and ventrally as development progressed (Figures 5E1–E3), as observed with other visual opsin profiles. For both *rh2* and *lws* opsin classes, expression patterns before first feeding were located proximal to the retinal pigment epithelium (Figures 5D3, E3).

## Discussion

This study presents a comprehensive mapping of visual opsins in salmonids that have undergone an additional round of genome duplication compared to other teleosts. Consistent with the concept of salmonid rediploidization, several visual opsin genes have not been retrained after the Ss4R genome duplication. Nevertheless, salmonids have maintained a broad repertoire of diverse visual opsins, exhibiting at least one representative gene in each of the five main opsin classes. The investigation primarily focused on Atlantic salmon, revealing an advanced array of Rh2 and Lws photopigment genes sensitive to medium and long wavelengths, respectively. Molecular dynamic modeling and tuning site interrogation predictions of the spectral characteristics between opsin types showed the greatest range of variation to be within the Rh2 class of photopigments. Further, expression analyzes showed that opsin expression is dynamic during development, with the *rh1-1* (traditionally responsible for scotopic vision) being the dominant visual opsin expressed post-hatching.

### The Complexity of Visual Opsins After Ss4R

The salmonid-specific fourth whole genome duplication, Ss4R, initially provided the common ancestor of all salmonids with a doubling of the complete genome sequence. However, subsequent Ss4R duplicate gene loss was predominated by pseudogenization (Berthelot et al., 2014; Lien et al., 2016) since the genomes of both Atlantic salmon and rainbow trout do not appear to contain the expected doubling of opsin genes compared to teleosts that did not undergo a 4R duplication event (e.g., northern pike) (Lin et al., 2017). In recent years, several genome assemblies of salmonids and a revised genome assembly of northern pike, Atlantic salmon, and rainbow trout have been deposited to Ensembl (Cunningham et al., 2018), which forms the basis here for analyzing salmonid genomes for the presence/absence of visual opsin genes. Indeed, this study identified a number of intact visual opsins, as well as several pseudogenes. As indicated in Lin et al. (2017) and Musilova et al. (2019), there are at least two genes in the Rh1 class for salmonids, but *rh1-2* paralogues have become pseudogenes. The current study showed that there is only one *sws2* gene in all salmonids analyzed and northern pike lack any orthologue of the *sws2* gene class (Lin et al., 2017). By comparison, multiple *rh1*, two *sws1* and two *sws2* paralogue genes were identified in *Cyprinus carpio* (common carp) and *Sinocyclocheilus* spp., i.e., fishes where the common ancestor also underwent a 4R event (Lin et al., 2017). Here, two *sws1* genes were found in all salmonids analyzed; however, in Atlantic salmon and brown trout the second copy of *sws1* seems to have undergone pseudogenization due to presence of indels (e.g., a 6 bp deletion) that would cause in frame protein truncations. The analyzes of the *rh2* opsin genes indicate that salmonids have 3–5 *rh2* genes; synteny analyses (Lin et al., 2017), and a close chromosomal location indicates that these *rh2* genes are tandem duplicated genes and not ohnologues. In northern pike four *rh2* genes are similarly located in an array on the same chromosome. Interestingly, four *rh2* genes were also identified in *C. carpio* and *Sinocyclocheilus* spp. but they do not localize to the same chromosome (Lin et al., 2017), suggesting that these genes either do not derive from a tandem duplication or that sufficient genomic rearrangements have occurred to reposition these *rh2* genes to different chromosomes. Phylogenetic analyzes revealed that *rh2-1*/*rh2-2* and *rh2-3*/*rh2-4* branch together, and as described in zebrafish (Chinen et al., 2003) sequence comparison showed a closer relation of *rh2-3* and *rh2-4* than *rh2-1* and *rh2-2*, indicating that tandem duplication of *rh2-3*/*rh2-4* occurred more recently. Coho salmon, rainbow trout and sockeye salmon have an additional *rh2-1* gene, with high sequence similarity, suggesting an even more recent duplication event in these species. Comparative analyzes of the *lws* opsin class showed that there are 3–4 genes in salmonids, with all species seeming to possess intact functional *lws*1 and *lws2* genes that are located close to each other on the same chromosome and with the *sws2* gene in proximity as shown in Lin et al. (2017). Conversely, several salmonid *lws3* and *lws4* genes are not identified at all, present as partial sequences, or has been assigned as a pseudogene. Notable, the updated genomes of Atlantic salmon and rainbow trout (Ensembl version 106) show that *lws3* and *lws4* are located on different chromosomes, while for the other salmonids under investigation *lws4* is not linked currently to a particular chromosome. By comparison, northern pike has four *lws* genes located on the same chromosome, where *lws1* and *lws2* and *lws3* and *lws4* form distinct clades. In contrast to salmonids, there are two *lws* genes in *C. carpio* and *Sinocyclocheilus* spp. located on different chromosomes (Lin et al., 2017).

### Spectral Properties of Atlantic Salmon Visual Photopigments

Spectral properties of visual opsins in several salmonids have already been analyzed by microspectrophotometry (MSP) of photoreceptors (Cheng et al., 2006), however the results do not take into account the diversity within the Rh2 and Lws classes. Here, manual interrogation of the Sws1, Rh1 and Lws photopigments (Musilova et al., 2019) and molecular dynamics simulations-based modeling approaches for the Sws2 and four Rh2 visual photopigment (Patel et al., 2018, 2020) were applied to estimate the spectral properties of all photopigments within the five opsin classes of Atlantic salmon. For Sws1 and Rh1, the manual interrogation gave λ_*max*_ of 360 and 509 nm, respectively, and were in close accordance with the MSP measurements at 361 and 515 nm (Cheng et al., 2006). The molecular dynamics simulations-based modeling of Sws2 gave a λ_*max*_ of 420 nm while MSP measurements gave a λ_*max*_ 435 nm (Cheng et al., 2006). Notably, the spectral sensitivity of a photopigment can be altered by a vitamin A_1_ to A_2_ shift of the chromophore, where the A_2_ give higher λ_*max*_ than A_1_ (Bridges, 1972; Hárosi, 1994) and altered ratio of A_1_/A_2_ has been shown to vary with temperature and daylength in coho salmon (Temple et al., 2006). However, the MSP results indicated that the retinas were primarily based on vitamin A_1_ (Cheng et al., 2006) and in the molecular dynamics simulations-based modeling approaches for the Sws2 and four Rh2 the λ_*max*_ used a standard A_1_-based rhodopsin template, indicating that the 15 nm difference in the λ_*max*_ for Sws2 is not due to different chromophores. When comparing MSP to molecular dynamics approaches, it is possible that modeling predictions are more divergent when estimating λ_*max*_ values for opsin protein sequences that are less phylogenetically related to the template sequence (e.g., comparing Sws2 to an Rh1 template vs. Rh2 compared to Rh1 that share a higher sequence identity and are more similar in λ_*max*_ values). The absorbance maximum determined for Rh2 and Lws were 518 and 578 nm, respectively (Cheng et al., 2006) while the diversity within the Rh2 and Lws classes determined by molecular dynamics simulations-based modeling spanned from 472 to 511 and 546 to 560 nm, respectively. These results indicate that the MSP measurements were done on photoreceptor cells with Rh2-3 or Rh2-4 photopigments for the Rh2 class and Lws1 for the Lws class. Further, the 40 nm range within the Rh2 class of photopigments are in accordance with zebrafish where the four Rh2 photopigments span from 467 to 505 nm (Chinen et al., 2003; Patel et al., 2018), but the range between the Rh2-1/Rh2-2 and Rh2-3/Rh2-4 branches is greater in Atlantic salmon. As proposed in agnathans, alterations in gene copies and subsequent mutations play an important role in evolution of color vision (Davies et al., 2009). Mutagenesis studies have shown that amino acid position 122 in teleost Rh2 opsins is essential in determining green-shifted λ_*max*_ (>495 nm) when occupied by Glu (E), and blue-shifted λ_*max*_ (<495 nm) when occupied by Gln (Q). This E122Q substitution alone accounts for ∼15 nm of determined spectral shift (Chinen et al., 2005; Patel et al., 2018). Supplementary Figure 4 shows that Atlantic salmon Rh2-1 and Rh2-2 have Q122 whereas Rh2-3 and Rh2-4 have E122. Which likely explains the blue vs. green spectral shift among salmonid Rh2 photopigments. The absorbance maximum of visual opsins in the anadromous Atlantic salmon range from 360 to 569 nm with representatives in all five opsin classes, providing specialized vision for living both in rivers and in the open ocean. In comparison, the benthopelagic Atlantic cod (*Gadus morhua*) lack representatives in the Sws1 and Lws classes, but has a greater repertoire within the Sws2 and Rh2 classes (Valen et al., 2014).

### Differential Gene Expression Profiles

Color visual capacity can be determined by several mechanisms, including changes in the type of chromophore used and/or the opsin expressed, the latter of which is influenced by opsin gene duplication or loss, as well as by changes in expression levels as a function of development or change of habitat (Carleton et al., 2020). For example, the gain and loss of UV-sensitive cones in salmonids are well studied in relation to their sea-river migration paths (Allison et al., 2003, 2006; Cheng et al., 2006). Previously, a two-step developmental profile of the duplex retina in Atlantic cod was demonstrated, where development of scotopic vision was concurrent with life-stage transition and diminishing expression of some of the *rh2* genes (Valen et al., 2016). In Atlantic salmon, with a more direct developmental process, the results of this study are consistent with previous findings showing that the salmon retina is duplex from early developmental stages (Cheng et al., 2007) and that rod opsin is the dominantly expressed visual opsin class post-hatching. As described in Chinook salmon (Cheng et al., 2007), the results presented here revealed that early opsin expression in the retina is located centrally, but expands dorsally and ventrally as eye development progresses. For example, *sws1* expression is topographically widely distributed before first feeding, which is in strong contrast to the restricted *sws1* expression profile close to the ciliary marginal zone at the smolt stage of rainbow trout (Allison et al., 2003). The number and distribution of *sws1* expressing cones in the whole retina at early stages indicates that UV light is likely to be very important for vision in rivers and lakes during early development. Unlike the marine larvae of Atlantic halibut (*Hippoglossus hippoglossus*) where *sws1* expression is restricted to the ventral part of the retina where it detects downwelling UV light, the distribution of *sws1* cones in the whole eye of Atlantic salmon suggests that UV light is detected from all directions (Helvik et al., 2001).

Due to high sequence identity between opsin genes within Rh2 and Lws classes, respectively, RNA *in situ* hybridization experiments were designed not to distinguish between different paralogues genes, but for opsin genes from within a class. However, RNA sequencing analyzes were able to resolve specific expression profiles for opsin genes within a class, where opsins were shown to be differentially expressed during development, with important functional implications. For example, *rh2-3* and *rh2-4*, resulting in photopigments that maximally absorb wavelengths above 500 nm, were more highly expressed at first feeding than *rh2-1* and *rh2-2*, with photopigments that were predicted to have λ_*max*_ 471 and 475 nm. Similarly, the expression levels of *lws1* and *lws3* (with photopigments that maximally absorb at λ_*max*_ 546 and 560 nm, respectively) are minimal compared to the expression of *lws2* and *lws4* (both photopigment types predicted to have a λ_*max*_ value at 553 nm). These patterns of differential expression might be a result of distinct expression of opsins within a class during life history transitions, as observed for *sws1* (Allison et al., 2003). As such, further studies that analyze the expression profiles of opsins within both Rh2 and Lws classes through smoltification would be of great scientific and commercial interest. In conclusion, this study describes the opsin repertoire of several species of salmonids, accompanied by more in depth analyzes of spectral tuning and expression profiles of Atlantic salmon photopigment genes.

## Data Availability Statement

The sequences of Atlantic salmon visual opsin genes can be found at the National Center for Biotechnology Information (NCBI) GenBank repository, https://www.ncbi.nlm.nih.gov/genbank/, ON456431–ON456441.

## Ethics Statement

Animal facilities, husbandry and sampling procedures were conducted in accordance with Norwegian Food Safety Authority recommendations.

## Author Contributions

ME did the *in silico* analyzes of the genomes for visual opsins, designed the primers for molecular cloning, did the interpretation of the RNA sequencing data and phylogenetic analyzes, performed the RNA *in situ* hybridization, made figures, and wrote the manuscript. WD and JM interpreted the complexity of the visual opsins, did the phylogenetic analyzes and the manual interrogation of spectral properties, made figures, and wrote and revised the manuscript. DP, JB, DS, and JP designed and performed the spectral modeling of the visual opsins, made figures, and revised the manuscript. RK did the molecular cloning and made the RNA probes. JH designed the study, interpreted the complexity of the visual opsins, the phylogenetic analyzes, RNA sequencing and *in situ* hybridization results, and revised the manuscript. All authors contributed to the article and approved the submitted version.

## Conflict of Interest

The authors declare that the research was conducted in the absence of any commercial or financial relationships that could be construed as a potential conflict of interest.

## Publisher’s Note

All claims expressed in this article are solely those of the authors and do not necessarily represent those of their affiliated organizations, or those of the publisher, the editors and the reviewers. Any product that may be evaluated in this article, or claim that may be made by its manufacturer, is not guaranteed or endorsed by the publisher.

## Supplementary Material

Supplementary Figure 1 Amino acid alignment of Rh1-1.

Supplementary Figure 2 Amino acid alignment of Sws1-1 and Sws1-2.

Supplementary Figure 3 Amino acid alignment of Sws2.

Supplementary Figure 4 Amino acid alignment of Rh2-1, Rh2-2, Rh2-3, and Rh2-4.

Supplementary Figure 5 Amino acid alignment of Lws1, Lws2, Lws3, and Lws4.

Supplementary Figure 6 Nucleotide alignment of *rh1-2.1* and *rh1-2.2.*

Supplementary Figure 7 Nucleotide alignment of *sws1-2.*

Supplementary Figure 8 Extended images of visual opsin expression in the developing Atlantic salmon eye. **(A1–E1)** Visual opsins after hatching (555 dd) and **(A2–E2)** before first feeding (720 dd). Scale bar 200 μm.

Supplementary Table 1 Primers used for molecular cloning of visual opsins in Atlantic salmon.

Supplementary Table 2 IDs and chromosome location of visual opsins in salmonids and northern pike.

Supplementary Table 3 Individual normalized count showing the expression of visual opsins in Atlantic salmon during development.
