# Supplementary figures and images for: An EvoDevo Study of Salmonid Visual Opsin Dynamics and Photopigment Spectral Sensitivity
Source: Front Neuroanat. 2022 Jul 11;16:945344. doi: 10.3389/fnana.2022.945344 (PMC9309310; doi:10.3389/fnana.2022.945344)

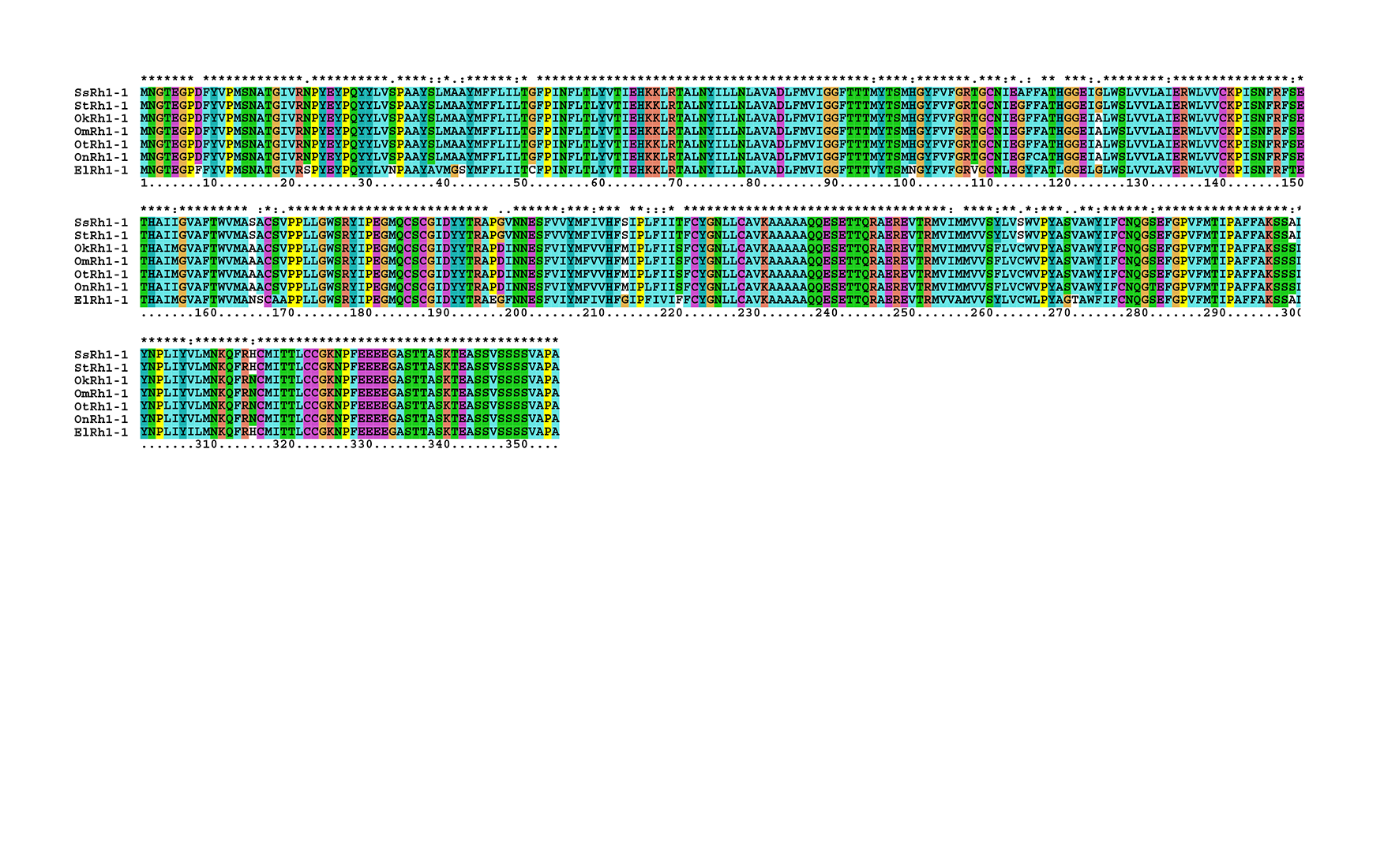

Supplement: Supplementary Figure 1 — Amino acid alignment of Rh1-1. [file Image_1.TIF]

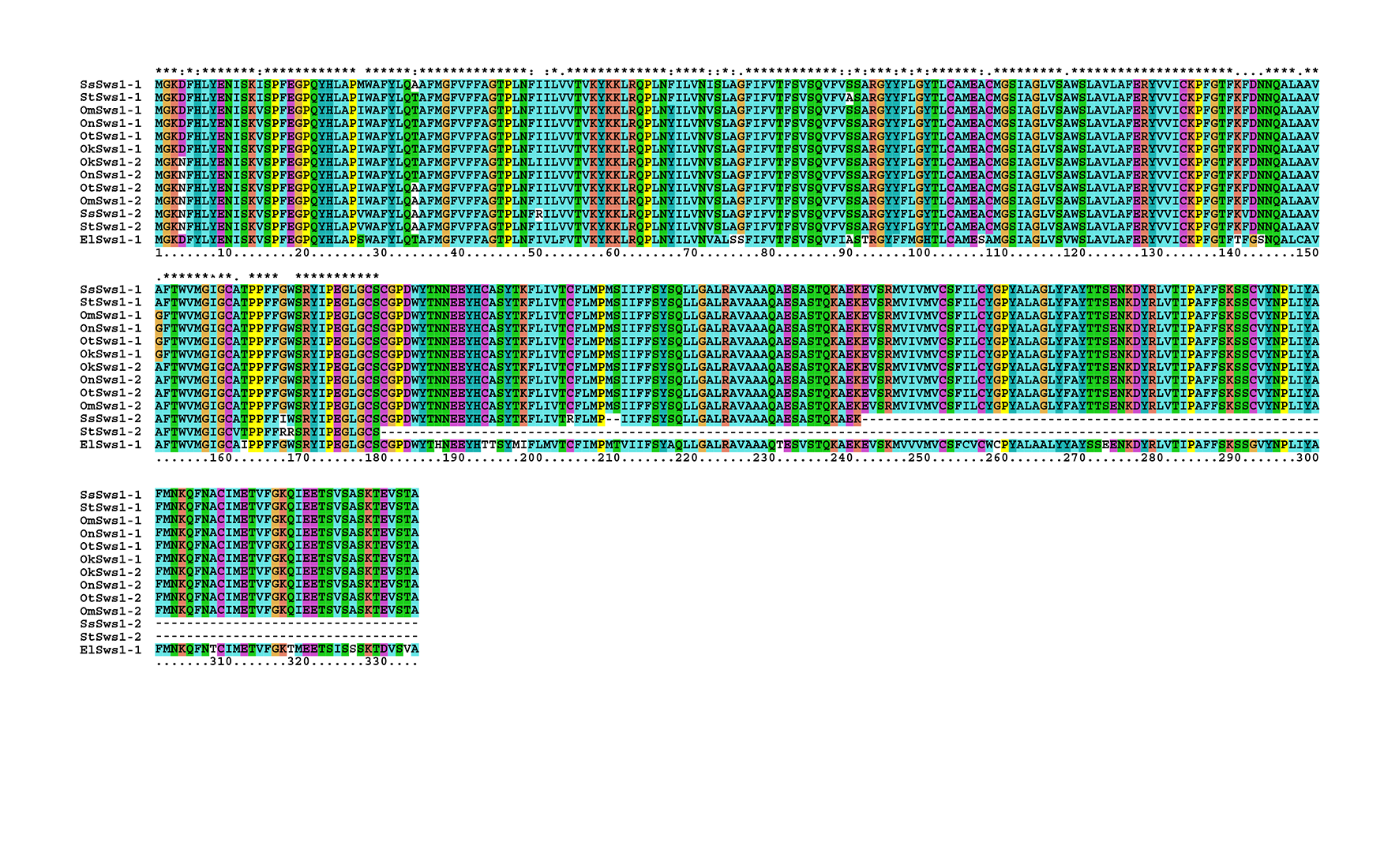

Supplement: Supplementary Figure 2 — Amino acid alignment of Sws1-1 and Sws1-2. [file Image_2.TIF]

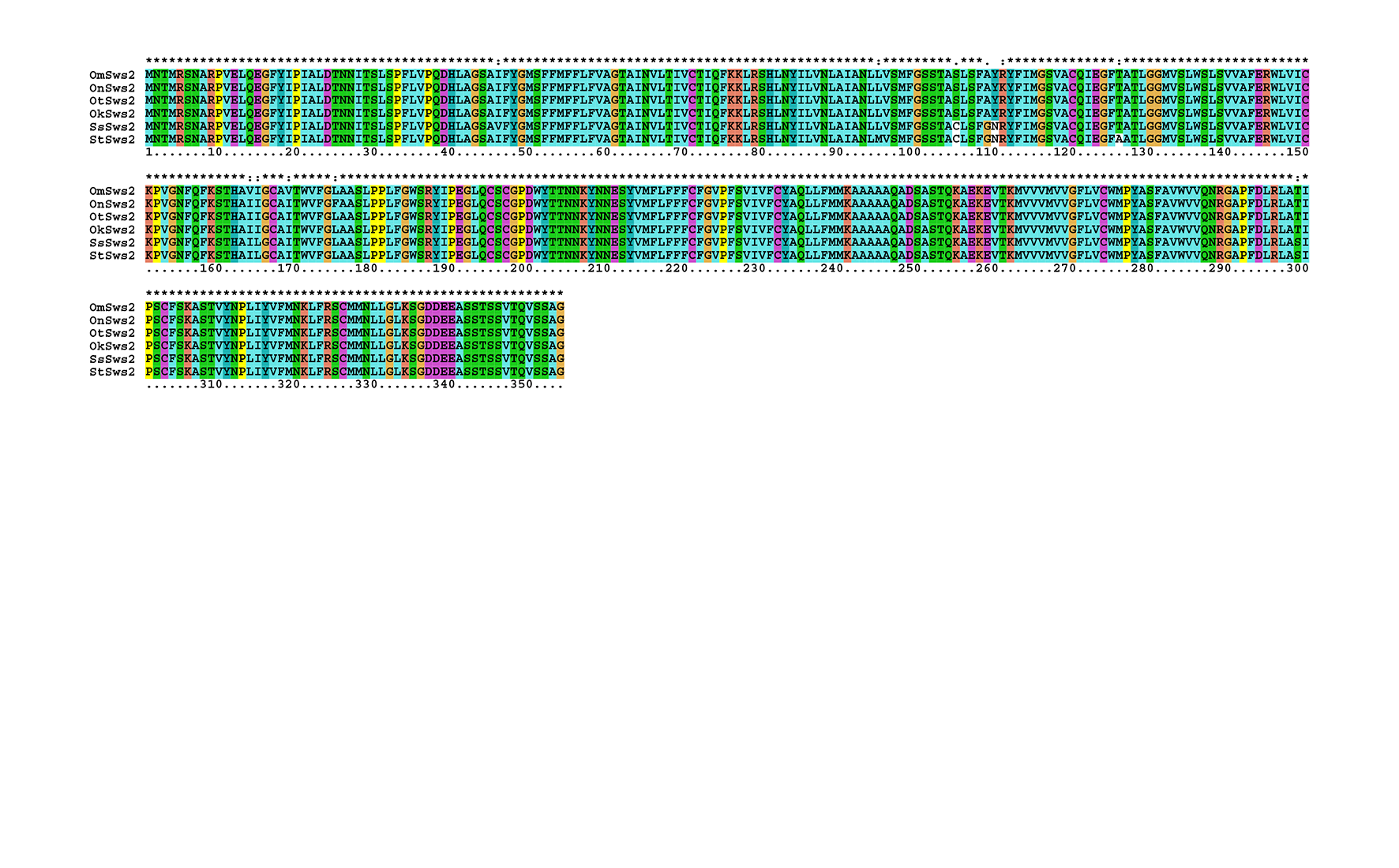

Supplement: Supplementary Figure 3 — Amino acid alignment of Sws2. [file Image_3.TIF]

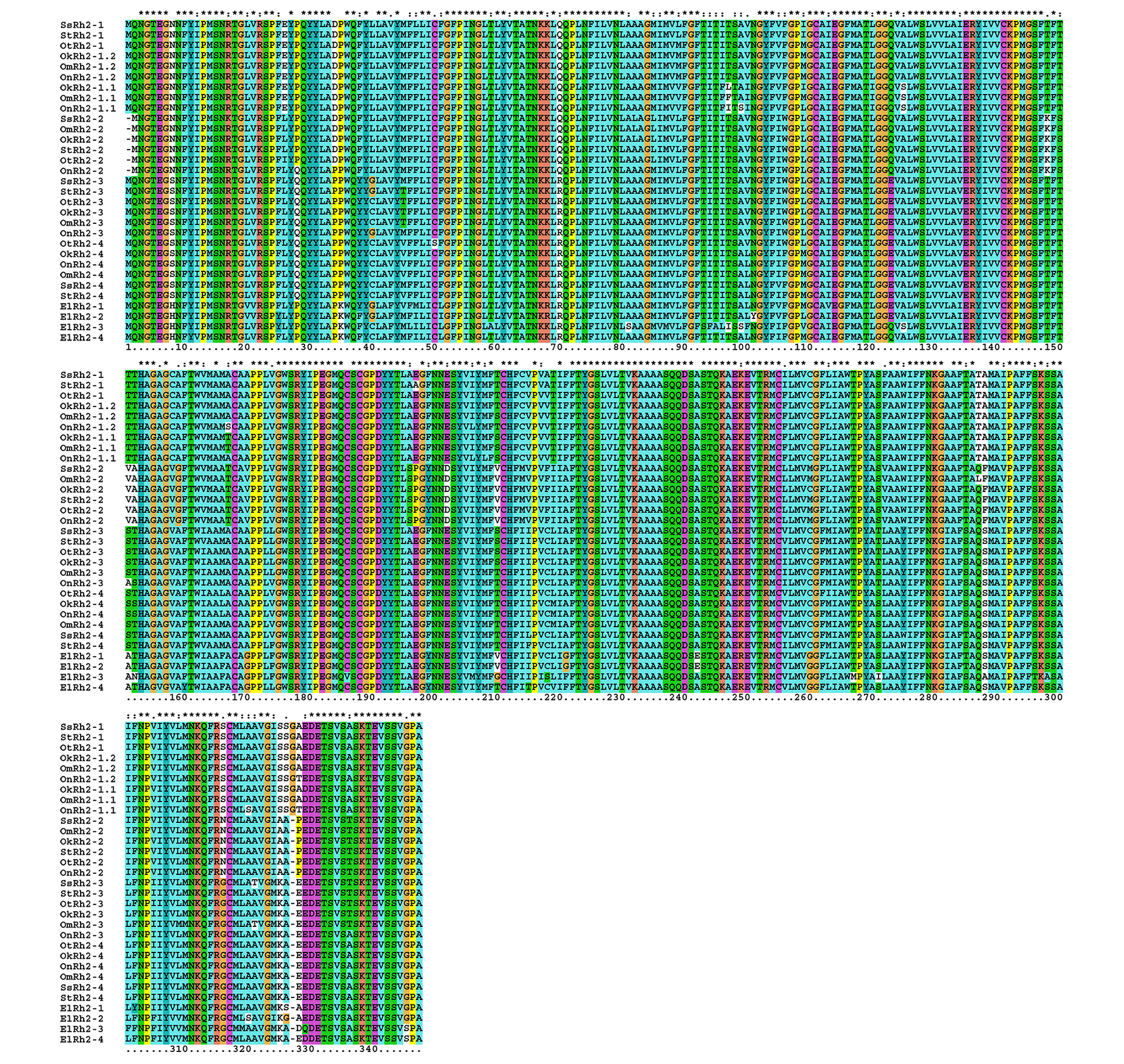

Supplement: Supplementary Figure 4 — Amino acid alignment of Rh2-1, Rh2-2, Rh2-3, and Rh2-4. [file Image_4.TIF]

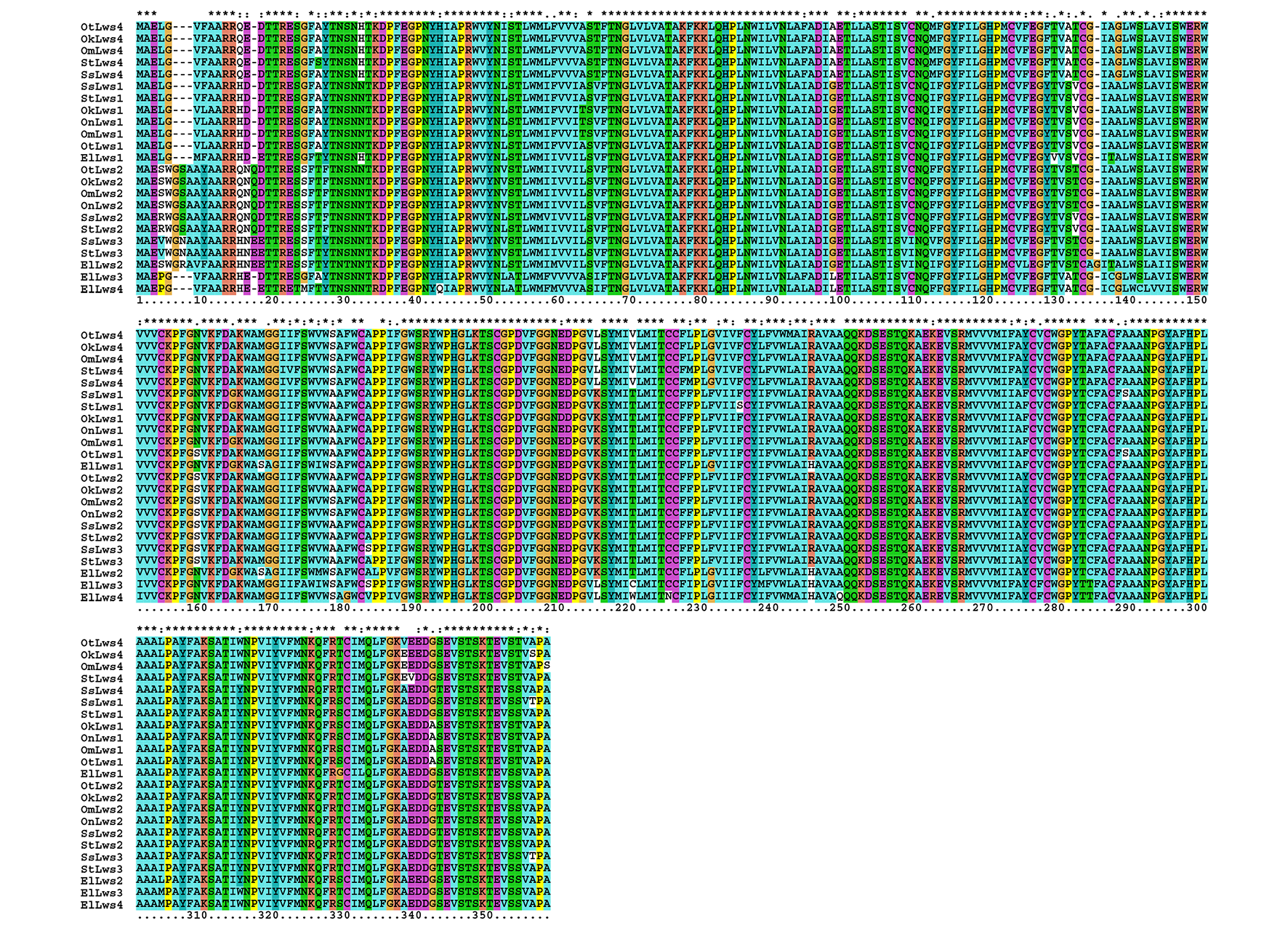

Supplement: Supplementary Figure 5 — Amino acid alignment of Lws1, Lws2, Lws3, and Lws4. [file Image_5.TIF]

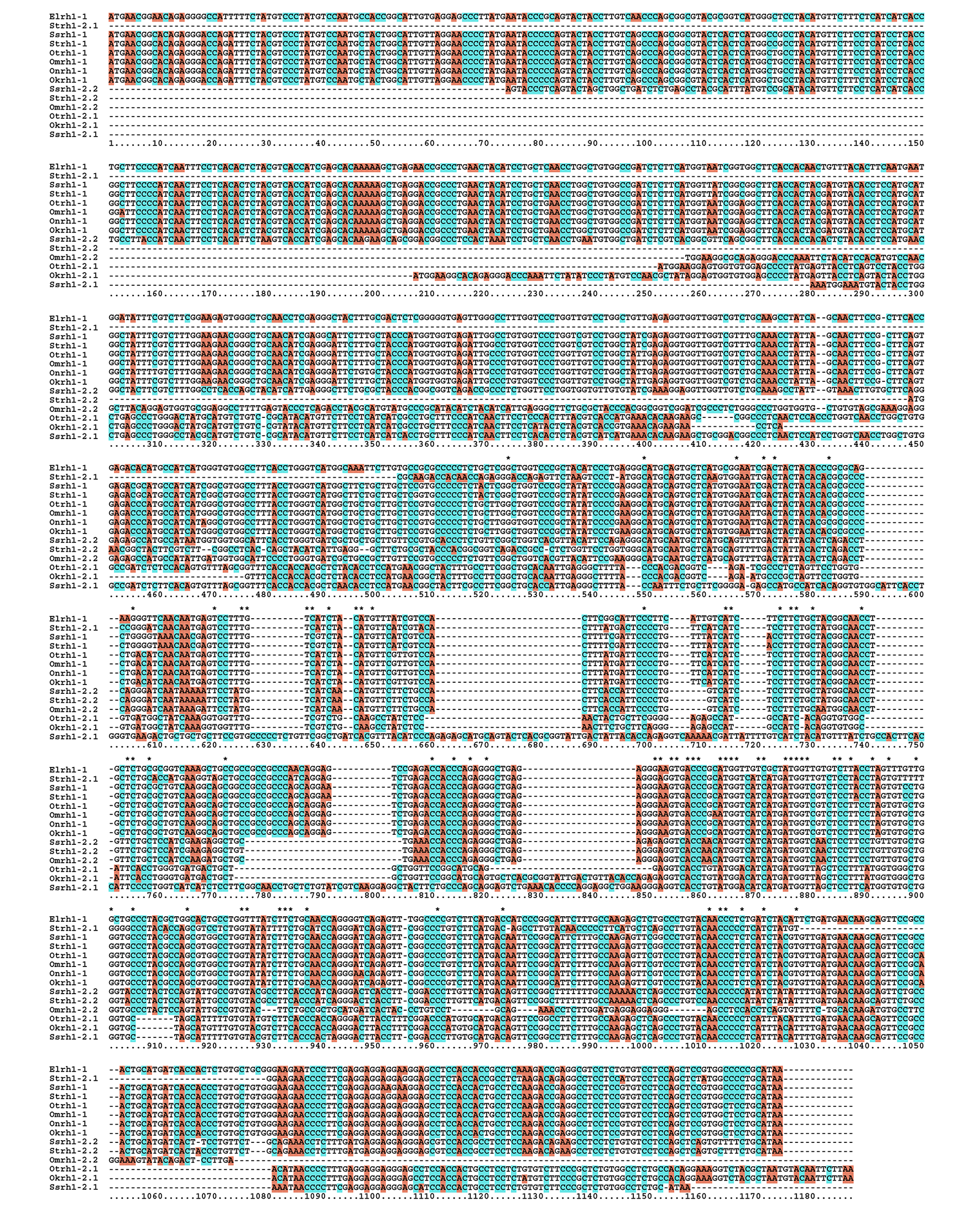

Supplement: Supplementary Figure 6 — Nucleotide alignment of rh1-2.1 and rh1-2.2. [file Image_6.TIF]

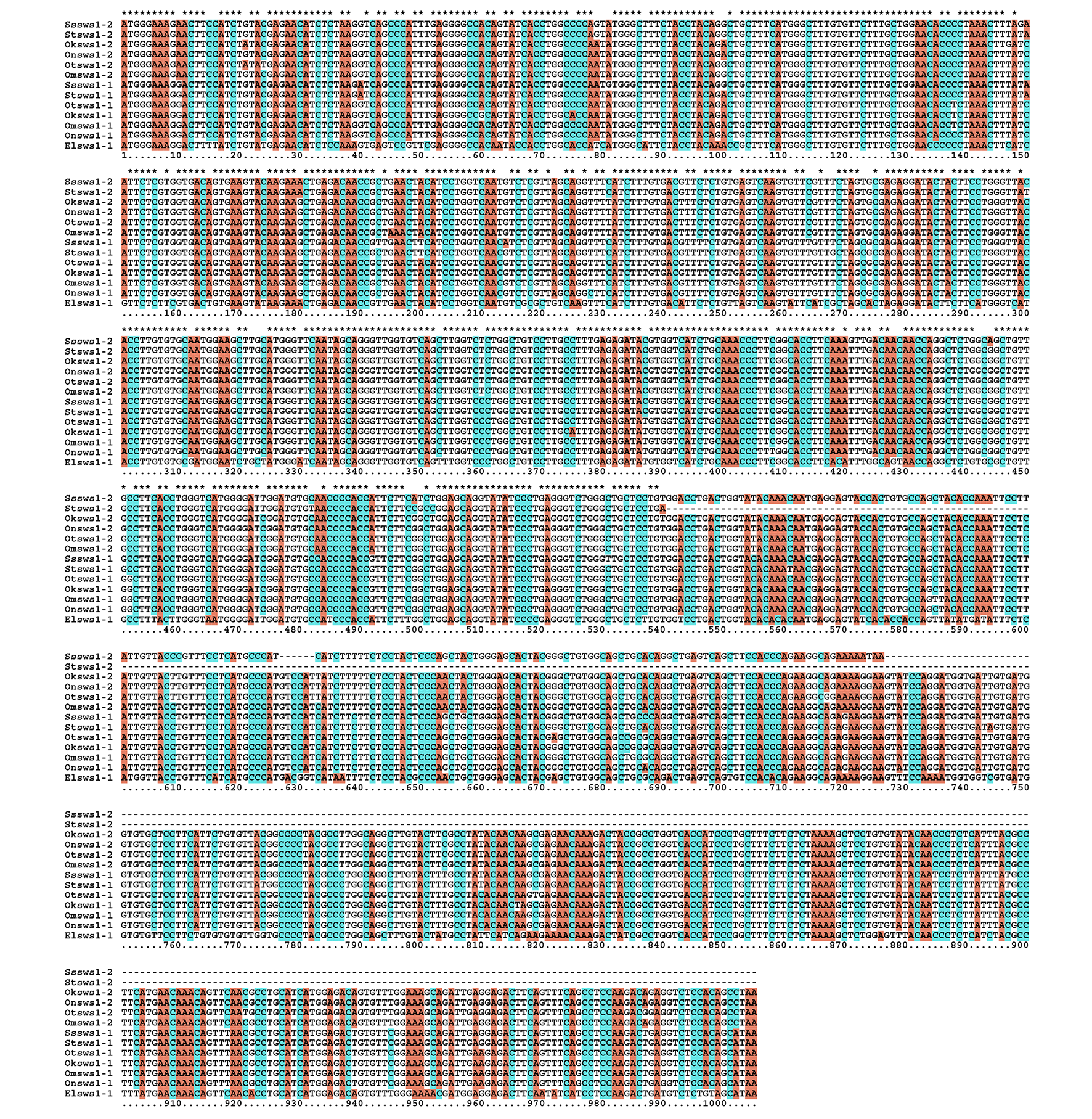

Supplement: Supplementary Figure 7 — Nucleotide alignment of sws1-2. [file Image_7.TIF]

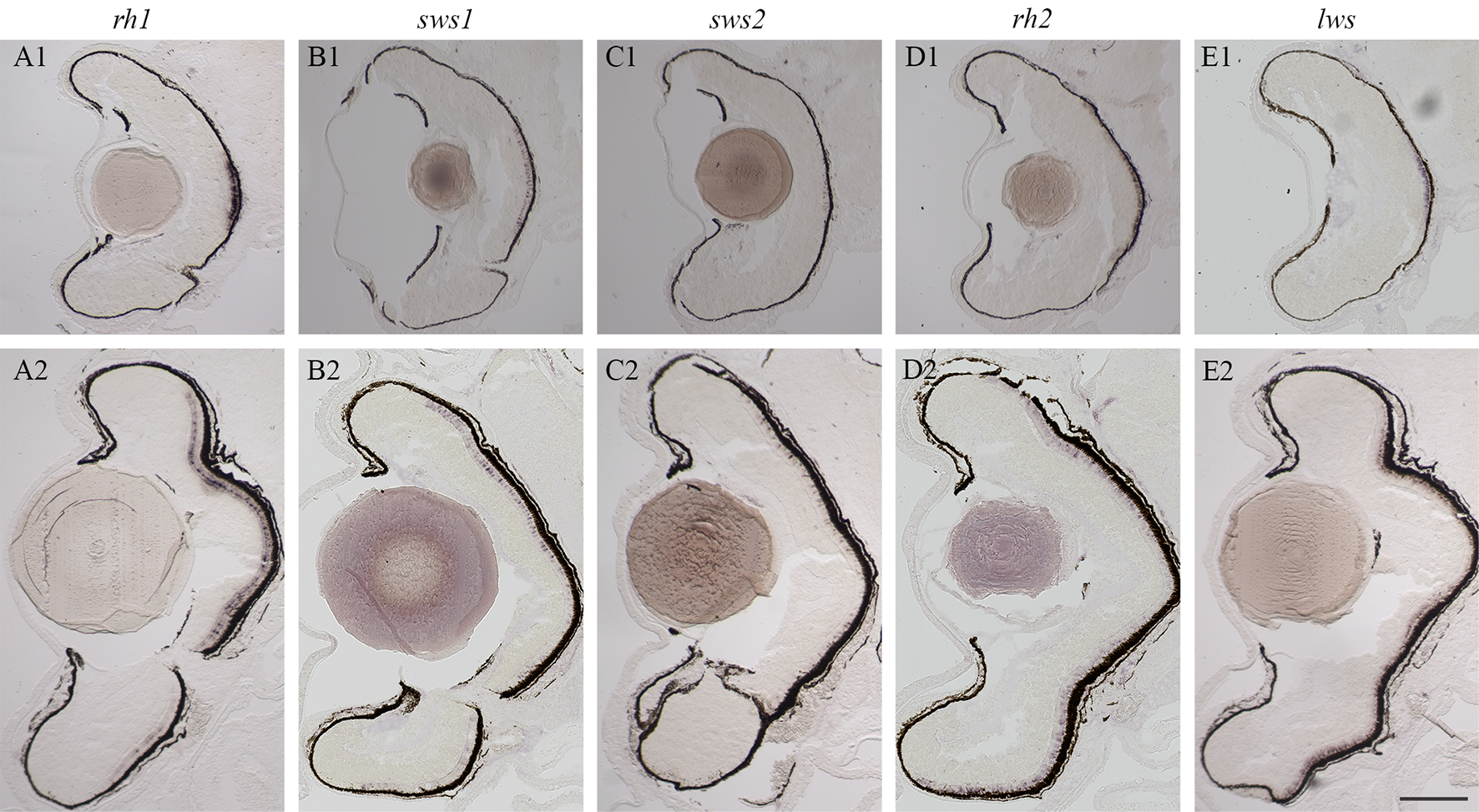

Supplement: Supplementary Figure 8 — Extended images of visual opsin expression in the developing Atlantic salmon eye. (A1–E1) Visual opsins after hatching (555 dd) and (A2–E2) before first feeding (720 dd). Scale bar 200 μm. [file Image_8.TIF]
